# Resistance Dynamics in a Romanian Critical Care Unit: Four Years of ESKAPE Pathogen Surveillance

**DOI:** 10.3390/medicina61122114

**Published:** 2025-11-27

**Authors:** Mihai Sava, Ioana Roxana Codru, Alina Simona Bereanu, Oana Stoia, Bogdan Ioan Vintila

**Affiliations:** 1 County Clinical Emergency Hospital of Sibiu, 550245 Sibiu, Romania; mihai.sava@ulbsibiu.ro (M.S.); oana.stoia@ulbsibiu.ro (O.S.); bogdan.vintila@ulbsibiu.ro (B.I.V.); 2 Faculty of Medicine, Lucian Blaga University of Sibiu, 550024 Sibiu, Romania

**Keywords:** ESKAPE pathogens, antimicrobial resistance, intensive care unit, resistance trends

## Abstract

*Background and Objectives:* Antimicrobial resistance is one of the most significant threats to modern healthcare, especially in intensive care units where ESKAPE pathogens*—Enterococcus faecium*, *Staphylococcus aureus*, *Klebsiella pneumoniae*, *Acinetobacter baumannii*, *Pseudomonas aeruginosa*, and *Enterobacter* spp.—account for the majority of healthcare-associated infections. Romania is among the European countries with the highest rates of antimicrobial consumption and resistance. This study aimed to describe the epidemiological trends and antimicrobial resistance profiles of ESKAPE isolates over a four-year period (2021–2024) in a Romanian ICU (Intensive Care Unit). *Materials and Methods:* We conducted a retrospective observational study of all microbiological samples collected from adult ICU patients at the Clinical Emergency County Hospital of Sibiu between 2021 and 2024. Data were extracted from the electronic laboratory system and included patient demographics, specimen types, isolated microorganisms, and antimicrobial resistance classifications. Statistical analyses were performed using Python libraries, with significance set at *p* < 0.05. *Results:* A total of 801 infections were recorded, of which 562 (70.2%) involved ESKAPE pathogens. The predominant organisms identified were *Klebsiella pneumoniae* (42.8%) and *Acinetobacter baumannii* (36.0%), followed by *Pseudomonas aeruginosa* (11.2%). Nearly half of the isolates (47.3%) were multidrug-resistant, and 22.3% were extensively drug-resistant. Respiratory specimens, particularly tracheal aspirates, accounted for the majority of the isolates and exhibited the highest proportion of resistant phenotypes. A significant temporal increase in extensively drug-resistant isolates was observed over the study period (*p* < 0.05). *Conclusions:* ESKAPE pathogens remain the leading causes of ICU infections in Romania, with *Klebsiella pneumoniae* and *Acinetobacter baumannii* contributing significantly to the burden of multidrug- and extensively drug-resistant infections. Strengthening infection prevention strategies, optimizing antimicrobial stewardship, and implementing continuous microbiological surveillance are essential to mitigate the evolving resistance crisis in Romanian critical care settings.

## 1. Introduction

The global ESKAPE group bacteria—comprising *Enterococcus faecium, Staphylococcus aureus*, *Klebsiella pneumoniae*, *Acinetobacter baumannii*, *Pseudomonas aeruginosa*, and *Enterobacter* spp.—represents a group of highly virulent and adaptive bacterial pathogens responsible for the majority of hospital-acquired infections. These organisms are known for their ability to acquire resistance, which allows them to avoid multiple classes of antimicrobial agent. Standard treatment regimens are becoming less effective, and bacteria are adapting to survive in modern healthcare settings. While recent years have seen the approval of new antimicrobial agents and β-lactamase inhibitor combinations aimed at targeting resistant Gram-negative bacteria, ESKAPE pathogens continue to present significant therapeutic challenges [1,2,3,4,5].

Healthcare-associated infections are a significant problem in hospitals, and strict infection control is vital to prevent them. Important HAIs (Healthcare-Associated Infections) include infections associated with antibiotic use, such as *Clostridioides difficile* enterocolitis, and those caused by bacteria in intensive care units, such as the members of the ESKAPE group. HAIs increase patient morbidity and mortality and can be caused by factors like invasive procedures and medical devices [6,7,8].

Critically ill patients admitted to intensive care units present unique risk factors that predispose them to infections with multidrug-resistant ESKAPE pathogens. Prolonged ICU stays, frequent exposure to broad-spectrum antibiotics, high-risk surgical interventions, and the use of invasive monitoring and life-support devices—such as mechanical ventilation, central venous catheters, and urinary catheters—create a suitable environment for both colonization and infection by resistant organisms. Furthermore, underlying chronic conditions, including malignancies, diabetes mellitus, and chronic obstructive pulmonary disease, and a weak baseline immune system increases susceptibility to resistant infections. The management of infections in this population is further complicated by alterations in pharmacokinetics and pharmacodynamics due to organ dysfunction, systemic inflammation, and fluid shifts, which collectively alter optimal antimicrobial dosing and therapeutic efficacy. In this context, ESKAPE pathogens exploit both host vulnerabilities and antimicrobial pressure, contributing to persistent infections, treatment failure, and increased mortality [9,10,11,12,13,14].

Romania is one of the highest consumers of antibiotics within the European Economic Area, with a defined daily dose of 25.7 per 1000 inhabitants in 2021, significantly higher than the European average of 16.4. This excessive use of antimicrobials is linked to some of the highest levels of antimicrobial resistance in Europe. Several systemic factors contribute to this situation, including the widespread use of broad-spectrum agents, inadequate implementation of antimicrobial stewardship programs, limitations in hospital infection control infrastructure, biofilm formation, and a shortage of trained specialists in infectious disease and microbiology [15]. Additionally, the lack of systematic local surveillance data on antimicrobial consumption, healthcare-associated infections, and resistance patterns interferes with the ability to establish evidence-based treatment guidelines and targeted infection prevention measures. Despite recent national initiatives aimed at improving antibiotic regulation and increasing public awareness, without continuous surveillance and stewardship efforts, infections caused by highly resistant organisms, particularly *Klebsiella pneumoniae* and *Acinetobacter baumannii*, may become untreatable. This could endanger the safety and effectiveness of advanced medical care, including oncology, surgery, and intensive care medicine [16,17,18].

The increasing prevalence of multidrug-resistant ESKAPE pathogens, combined with the limited availability of region-specific data, highlights the urgent need for local surveillance to guide clinical decision-making in high-risk settings, such as the ICU. The global rise of ESKAPE organisms as priority pathogens emphasizes the importance of understanding their epidemiological trends in healthcare systems with varying resources, such as those found in Romania [19,20,21,22].

The objective of this study is to analyze the epidemiological trends and antimicrobial resistance profiles of ESKAPE isolates collected over a four-year period (2021–2024) in a Romanian ICU. The findings aim to enhance infection control strategies, inform empirical antimicrobial therapy, and strengthen local and regional antimicrobial stewardship efforts.

## 2. Materials and Methods

We conducted a retrospective observational study analyzing all microbiological samples collected in the adult Intensive Care Unit of the Clinical Emergency County Hospital in Sibiu between 2021 and 2024, with a specific focus on pathogens from the ESKAPE group.

### 2.1. Data Collection

Data were extracted from the electronic laboratory information system and anonymized before analysis to ensure patient confidentiality. For each positive microbiological culture, the following variables were recorded: year of isolation, patient age and sex, specimen type (e.g., blood, urine, respiratory, wound), the identified microorganism, and the antimicrobial resistance classification as reported by the microbiology laboratory.

### 2.2. Statistical Analysis

Descriptive statistics were reported as mean ± standard deviation (SD) or median with interquartile range (IQR) for continuous variables, and as frequencies with corresponding percentages for categorical variables. Group comparisons were performed using the Chi-square test or Fisher’s exact test for categorical variables, and using the Kruskal–Wallis test for continuous variables. Temporal trends were assessed by examining annual distributions of the most frequently isolated organisms and their associated antimicrobial resistance categories. Statistical significance was defined as a *p*-value < 0.05. All analyses were conducted using Python v. 3.12, employing libraries such as pandas, scipy, and matplotlib.

## 3. Results

**Figure 1:**
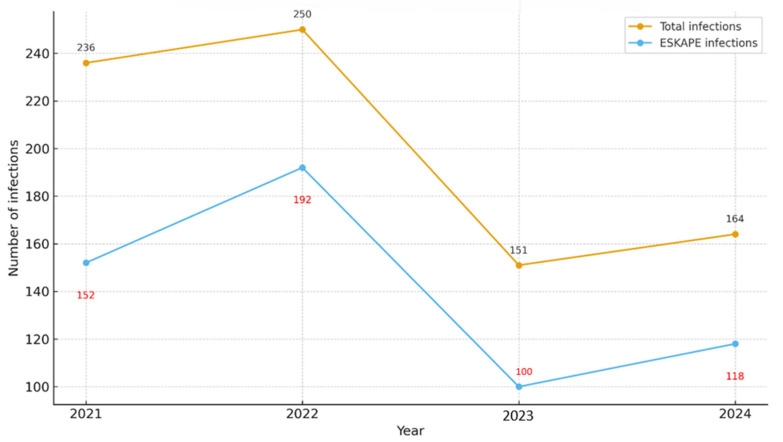
Yearly distribution of Total vs. ESKAPE infections.

Between 2021 and 2024, a total of 801 infections were documented in the adult ICU. Of these, 562 cases (70.2%) were attributed to pathogens from the ESKAPE group. The annual distribution of these infections is summarized in Table 1 and illustrated in Figure 1. In 2021, a total of 236 infections were documented, of which 152 (64.4%) were attributable to ESKAPE pathogens. In 2022, both the overall infection count and the absolute number of ESKAPE-related cases increased, reaching 250 and 192, respectively, with ESKAPE organisms accounting for the highest annual proportion (76.8%). In 2023, the total number of infections declined to 151, including 100 ESKAPE cases (66.2%). In 2024, 164 infections were recorded, of which 118 (72.0%) were caused by ESKAPE pathogens.

- Study Population

Between 2021 and 2024, a total of 573 positive microbiological cultures were identified in the ICU. The mean patient age was 66.2 ± 36.3 years, with a median of 66 years (IQR: 54–75). Among these cases, 341 (59.5%) were male and 221 (38.6%) were female. The most frequently collected specimen types were tracheal aspirates (278; 48.5%), followed by surgical wound, abscess, or ulcer samples (71; 12.4%) and blood cultures (67; 11.7%) (Table 2).

- Gender-specific distribution of isolates

Analysis of ESKAPE pathogens stratified by patient sex revealed a statistically significant difference in organism distribution (χ^2^ = 11.4, df = 5, *p* = 0.043). Although *Klebsiella pneumoniae* and *Acinetobacter baumannii* remained the predominant species in both genders, minor gender differences were observed in *Pseudomonas aeruginosa* and *Staphylococcus aureus.*

Conversely, the distribution of antimicrobial resistance categories did not differ significantly between male and female patients (χ^2^ = 3.9, df = 7, *p* = 0.79), with multidrug-resistant and extensively drug-resistant isolates proportionally represented across both groups.

- Microbiological Specimens

Microbiological isolates were obtained from diverse clinical specimens. The majority originated from respiratory samples, predominantly tracheal aspirates, which accounted for nearly half of all cultures.

The distribution of pathogens varied significantly across specimen types. *Acinetobacter baumannii* and *Klebsiella pneumoniae* were disproportionately isolated from respiratory specimens, consistent with their established role in ventilator-associated pneumonia. In contrast, *Staphylococcus aureus* was more frequently recovered from wound and abscess samples, reflecting its tropism for skin and soft tissue infections. Blood cultures yielded a smaller yet diverse set of isolates, with Gram-negative organisms predominating.

When resistance patterns were analyzed by specimen type, multidrug-resistant and extensively drug-resistant phenotypes were particularly prevalent among respiratory isolates. In contrast, blood and wound isolates exhibited greater heterogeneity in resistance profiles, although resistant phenotypes remained clinically significant (Table 3).

**Figure 2:**
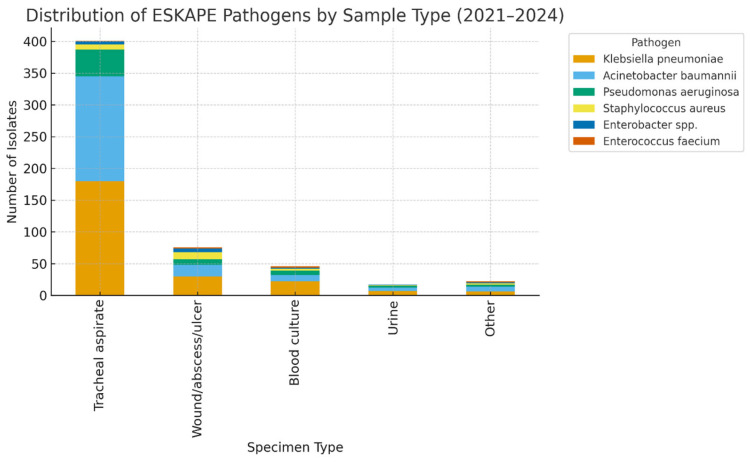
Distribution of ESKAPE Pathogens by Sample Type.

Association analysis revealed that the distribution of pathogens varied significantly by specimen type (χ^2^ = 3506.2, df = 176, *p* < 0.001), confirming that certain bacterial species exhibit a predilection for specific clinical sources. Similarly, antimicrobial resistance profiles differed significantly across specimen types (χ^2^ = 2923.8, df = 192, *p* < 0.001). Respiratory specimens were most frequently associated with MDR and XDR phenotypes, whereas blood and wound samples demonstrated a more heterogeneous distribution of resistance (Figure 2).

**Figure 3:**
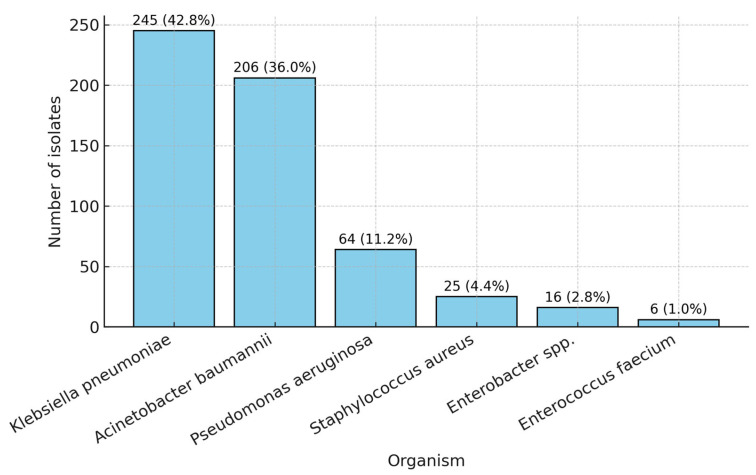
Distribution of the ESKAPE organisms (2021–2024).

The ESKAPE group accounted for the vast majority of ICU isolates throughout the study period. Within this group, *Klebsiella pneumoniae* was the most frequently identified species (245 isolates, 42.8%), followed by *Acinetobacter baumannii* (206 isolates, 36.0%) and *Pseudomonas aeruginosa* (64 isolates, 11.2%). Gram-positive members were less common, with *Staphylococcus aureus* accounting for 4.4% (25 isolates) and *Enterococcus faecium* accounting for 1.0% (6 isolates). *Enterobacter* spp. were detected in 16 cases (2.8%) (Table 4, Figure 3).

**Figure 4:**
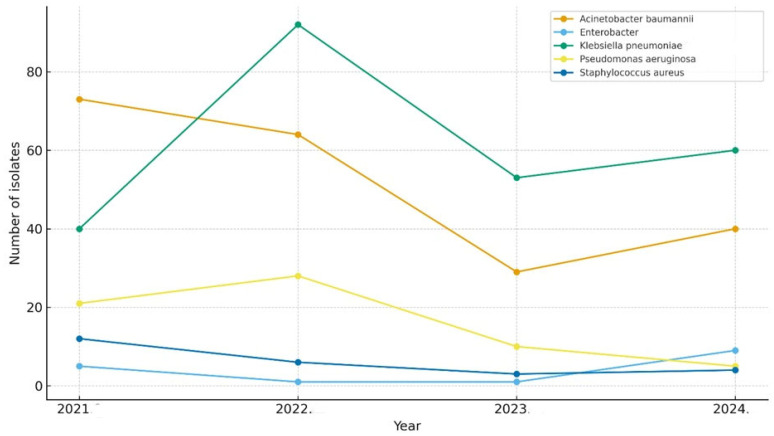
Yearly distribution of ESKAPE pathogens.

Figure 4 illustrates the annual distribution of ESKAPE pathogens isolated from ICU patients between 2021 and 2024. *Klebsiella pneumoniae* and *Acinetobacter baumannii* were the predominant organisms throughout the study period, accounting for the highest number of isolates each year. *Pseudomonas aeruginosa* and *Enterobacter* spp. were consistently isolated at lower frequencies, showing modest fluctuations over time. *Staphylococcus aureus* had the lowest isolation rate among the ESKAPE group, maintaining a stable, low rate across all 4 years.

- Antimicrobial Resistance—ESKAPE group

**Figure 5:**
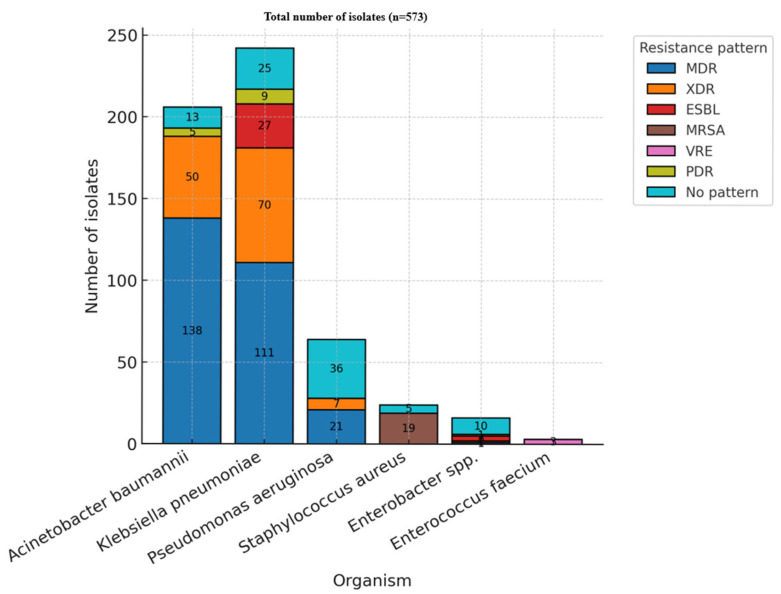
Resistance patterns—ESKAPE (MDR—multidrug-resistant; XDR—extensively drug-resistant; ESBL—extended-spectrum beta-lactamase; MRSA—methicillin-resistant *Staphylococcus aureus*; VRE—vancomycin-resistant *Enterococcus*; PDR—pan-drug resistant).

Resistance classifications follow the international definitions proposed by Magiorakos et al.: MDR—multidrug-resistant, XDR—extensively drug-resistant, and PDR—pan-drug-resistant [23]. Among ESKAPE organisms, nearly half of isolates (271, 47.3%) were classified as multidrug-resistant, while 128 isolates (22.3%) were extensively drug-resistant. A smaller proportion exhibited no specific resistance pattern (89 isolates, 15.5%) (Table 5 and Figure 5).

The distribution of antimicrobial resistance categories varied significantly among the different organisms analyzed (*p* < 0.05). *Acinetobacter baumannii* and *Klebsiella pneumoniae* represented the predominant sources of multidrug-resistant and extensively drug-resistant isolates, underscoring their established roles as key contributors to antimicrobial resistance in intensive care unit settings. *Pseudomonas aeruginosa* was also notably present among MDR cases. In contrast, resistance in *Staphylococcus aureus* was primarily attributed to methicillin resistance. Instances of pan-drug resistance were rare, observed only in a limited subset of *Acinetobacter* and *Klebsiella* isolates.

From a temporal standpoint, the prevalence of multidrug-resistant organisms remained consistently elevated throughout 2021–2024. In contrast, the proportion of extensively drug-resistant isolates increased relative to the latter years of the study.

- Temporal Trends in Resistance

Temporal analysis of resistance trends within the ESKAPE pathogen group demonstrated a sustained predominance of multidrug-resistant phenotypes over the four-year study period. MDR isolates consistently accounted for the most significant proportion of resistant organisms from 2021 to 2024. In contrast, extensively drug-resistant isolates exhibited a progressive increase in prevalence during the latter part of the study period. Conversely, extensively drug-resistant isolates demonstrated a noticeable upward trend in the later years of the study.

Statistical analysis revealed a significant association between the year of isolation and resistance category (χ^2^ test, *p* < 0.05), indicating a temporal shift in the distribution of resistance profiles. This trend was particularly evident in the increasing prevalence of extensively drug-resistant (XDR) pathogens in the later years of the study.

Collectively, these findings underscore the predominance of ESKAPE pathogens in the ICU microbiological landscape, with *Klebsiella pneumoniae* and *Acinetobacter baumannii* emerging as the principal contributors, and the persistently high prevalence of multidrug-resistant and extensively drug-resistant phenotypes, particularly in respiratory specimens.

## 4. Discussion

This four-year retrospective study provides a detailed overview of the epidemiological characteristics and antimicrobial resistance profiles of ESKAPE pathogens isolated from ICU patients in a tertiary Romanian hospital. Our findings reveal that *Klebsiella pneumoniae* and *Acinetobacter baumannii* were the dominant species, together accounting for nearly 80% of all isolates. This distribution mirrors global data, in which these two Gram-negative bacteria are major contributors to ICU-acquired infections and antimicrobial resistance [1,3,19].

The findings emphasize the dynamic epidemiology of ICU infections, with Gram-negative ESKAPE pathogens—particularly *Klebsiella pneumoniae* and *Acinetobacter baumannii*—maintaining dominance over time while exhibiting notable shifts in prevalence patterns.

### 4.1. Comparison with Previous Studies

The predominance of respiratory isolates, particularly those obtained from tracheal aspirates, highlights the significant incidence of ventilator-associated infections among critically ill patients in the ICU. This trend reflects the considerable burden of healthcare-associated pneumonia in intensive care settings, where the use of invasive mechanical ventilation is a major predisposing factor. The presence of endotracheal tubes disrupts the body’s natural airway defenses, encourages the accumulation of respiratory secretions, and facilitates biofilm formation. These conditions collectively create an environment that facilitates bacterial colonization and subsequent infection. As a result, ventilator-associated pneumonia contributes markedly to increased morbidity, prolonged ICU stays, and elevated antibiotic consumption. These factors present serious challenges for infection control efforts and the effective management of antimicrobial stewardship. Consistent findings from studies conducted in Romania and other regions further support that respiratory tract infections are a leading cause of nosocomial infections among critically ill populations worldwide. Given these observations, there is a clear need for rigorous preventive strategies. Key measures include strict adherence to ventilator care bundles, comprehensive surveillance of respiratory pathogens, and judicious antimicrobial use. Implementing these strategies is essential to mitigate the impact of ventilator-associated infections and reduce the overall burden of respiratory tract infections in critical care environments [7,9,11,22,24,25,26,27].

The elevated proportion of multidrug-resistant and extensively drug-resistant strains, particularly among *Acinetobacter baumannii* and *Klebsiella pneumoniae*, further compounds this problem. These pathogens, frequently isolated from respiratory specimens, have emerged as major etiological agents of VAP (Ventilator-Associated Pneumonia) and other healthcare-associated infections. Their capacity to acquire and disseminate resistance determinants—mostly carbapenemases—has led to a sharp decline in the efficacy of key therapeutic agents. The resulting increase in carbapenem-resistant isolates reflects a broader global trend, with significant implications for patient outcomes and infection control practices. The limited availability of effective antimicrobial options often necessitates the use of last-resort drugs such as colistin or tigecycline, which are associated with variable efficacy and potential toxicity. These findings highlight the urgent need for robust antimicrobial stewardship programs, continuous microbiological surveillance, and the implementation of evidence-based infection prevention measures to curb the spread of MDR and XDR organisms in critical care environments [4,14,19,28,29,30]. The progressive increase in XDR isolates observed in the latter years of the study highlights the dynamic and worsening resistance landscape within the ICU environment.

Similar national data have reported that Romania remains among the European countries with the highest antimicrobial consumption and resistance rates [16,17,18,22,31]. Systemic factors—including the liberal use of broad-spectrum antibiotics, inconsistent infection control implementation, and limited stewardship infrastructure—likely contribute to the persistence of resistant strains [17,18]. In particular, *A. baumannii* and *K. pneumoniae* are well-documented to persist on surfaces and medical devices, facilitating transmission and colonization in ICU settings [13,32].

### 4.2. Clinical Implications and Stewardship

The sustained high prevalence of MDR and XDR phenotypes highlights an urgent need for strengthening antimicrobial stewardship programs at the hospital level. Empirical therapy in ICUs should increasingly rely on local microbiological surveillance data to ensure both adequate initial coverage and resistance control. Rapid diagnostic testing and molecular surveillance could further improve early detection of resistance mechanisms and inform targeted therapy [29,30,33,34,35].

Enhanced infection prevention measures—including strict device management, proper disinfection protocols, and staff education—are also essential to reducing transmission. Integration of multidisciplinary approaches involving intensivists, infectious disease specialists, and microbiologists can optimize antibiotic selection, reduce inappropriate prescribing, and improve patient outcomes [19,36,37,38].

### 4.3. Temporal and Demographic Trends

The temporal variability observed in isolate distribution, particularly the pronounced peak in *Klebsiella pneumoniae* isolates during 2022, may be attributable to several interrelated factors. Fluctuations in patient case-mix—such as changes in the proportion of severely ill, mechanically ventilated, or immunocompromised individuals—can significantly influence the prevalence of specific pathogens in intensive care settings. In addition, variations in antimicrobial use patterns, including the increased reliance on broad-spectrum or empiric antibiotic therapy during periods of clinical uncertainty, may have exerted selective pressure favoring the proliferation of resistant *K. pneumoniae* strains. The ongoing impact of the COVID-19 pandemic may also have contributed, as resource constraints, staff shortages, and disruptions in infection prevention practices have been linked to temporary surges in healthcare-associated infections globally [9,32,39,40].

Despite minor sex-related differences in species distribution, the absence of significant variation in resistance categories between male and female patients suggests that antimicrobial resistance pressure operates largely independently of patient gender. This uniformity implies that key determinants of resistance—such as antibiotic exposure, invasive procedures, and environmental factors—exert a comparable influence across patient subgroups. As a result, interventions to combat antimicrobial resistance should focus on institutional and clinical practice factors rather than on demographic characteristics alone [41,42].

#### Limitations

This study has several limitations that may affect the interpretation and reliability of its findings. First, its retrospective, single-center design limits generalizability to other ICUs; as a result, the resistance patterns observed may not reflect those seen in different regions or hospital settings. Second, the absence of clinical outcome data precludes correlating infection with resistant pathogens with patient mortality or morbidity, making it difficult to assess the actual clinical impact of these infections. Third, the lack of molecular analysis of resistance genes prevents the identification of the specific genetic mechanisms driving resistance, thereby restricting understanding of transmission pathways and resistance evolution. Finally, variation in sampling frequency and diagnostic practices across years may have influenced the temporal distribution of isolates, potentially biasing trend analyses. To address these gaps, future studies should prospectively collect both microbiological and detailed clinical outcome data from multiple centers, enabling more robust correlation between resistance profiles and patient outcomes. Incorporating advanced methodologies, such as whole-genome sequencing, would provide deeper insight into the genetic mechanisms of resistance and elucidate transmission pathways within and between healthcare facilities. Such comprehensive multicenter studies would significantly enhance understanding of antimicrobial resistance dynamics and inform targeted interventions to optimize infection control and patient care.

## 5. Conclusions

This four-year surveillance study reinforces the ongoing and evolving challenge posed by ESKAPE pathogens in Romanian intensive care units. The primary organisms identified were *Klebsiella pneumoniae* and *Acinetobacter baumannii*, both of which exhibited high levels of being multidrug- and extensively drug-resistant, particularly in respiratory infections. The persistent presence of resistant strains indicates a need to improve infection control measures, antimicrobial stewardship, and antibiotic prescribing practices. To address these challenges, a coordinated national strategy is essential that should include continuous microbiological surveillance, optimized empirical therapy based on local data, and strengthened infection prevention and control measures. Enhancing collaboration among intensivists, microbiologists, and infectious disease specialists will be an important factor in mitigating the spread of resistance and preserving the effectiveness of current antimicrobial therapies.

## Figures and Tables

**Table 1:** Total ICU infections and ESKAPE infections by year.

Year	Total Infections	ESKAPE Infections	% ESKAPE of Total
2021	236	152	64.4%
2022	250	192	76.8%
2023	151	100	66.2%
2024	164	118	72.0%
Total	801	562	70.2%

**Table 2:** Demographics and baseline characteristics (N = 573).

Characteristic	N	%
Total isolates	573	
Age, mean (SD)	66.2 (36.3)	
Age, median (IQR)	66 (54–75)	
Sex: Male	341	59.5
Sex: Female	221	38.6
Sample type: Tracheal aspirate	278	48.5
Sample type: Surgical wound/abscess/ulcer	71	12.4
Sample type: Blood	67	11.7

**Table 3:** Distribution of ESKAPE pathogens by specimen type (2021–2024).

Specimen Type	*Klebsiella pneumoniae*	*Acinetobacter baumannii*	*Pseudomonas aeruginosa*	*Staphylococcus aureus*	*Enterobacter* spp.	*Enterococcus faecium*	Total (n, %)
Tracheal aspirate	180 (64.7%)	165 (80.1%)	42 (65.6%)	8 (32.0%)	5 (31.3%)	1 (16.7%)	401 (70.0%)
Wound/abscess/ulcer	30 (10.9%)	18 (8.7%)	9 (14.1%)	11 (44.0%)	6 (37.5%)	2 (33.3%)	76 (13.3%)
Blood culture	22 (8.0%)	10 (4.9%)	7 (10.9%)	3 (12.0%)	2 (12.5%)	2 (33.3%)	46 (8.0%)
Urine	7 (2.5%)	5 (2.4%)	3 (4.7%)	1 (4.0%)	1 (6.3%)	0 (0%)	17 (3.0%)
Other (catheter tips, fluids)	6 (2.2%)	8 (3.9%)	3 (4.7%)	2 (8.0%)	2 (12.5%)	1 (16.7%)	22 (3.7%)
Total (N = 573)	245 (42.8%)	206 (36.0%)	64 (11.2%)	25 (4.4%)	16 (2.8%)	6 (1.0%)	573 (100%)

**Table 4:** Distribution of ESKAPE organisms (N = 573).

Organism	n	%
*Klebsiella pneumoniae* ssp. *pneumoniae*	245	42.8
*Acinetobacter baumannii*	206	36.0
*Pseudomonas aeruginosa*	64	11.2
*Staphylococcus aureus*	25	4.4
*Enterobacter* spp.	16	2.8
*Enterococcus faecium*	6	1.0

**Table 5:** Resistance categories—ESKAPE.

Organism	MDR	XDR	ESBL	MRSA	VRE	PDR	No Pattern
*Acinetobacter baumannii*	138	50	–	–	–	5	13
*Klebsiella pneumoniae* ssp.	111	70	27	–	–	9	25
*Pseudomonas aeruginosa*	21	7	–	–	–	–	36
*Staphylococcus aureus*	–	–	–	19	–	–	5
*Enterobacter* spp.	1	1	3	–	–	1	10
*Enterococcus faecium*	–	–	–	–	3	–	–

## Data Availability

Data are contained within this article.
